# Nano-Pesticides and Fertilizers: Solutions for Global Food Security

**DOI:** 10.3390/nano14010090

**Published:** 2023-12-28

**Authors:** Yuying Tang, Weichen Zhao, Guikai Zhu, Zhiqiang Tan, Lili Huang, Peng Zhang, Li Gao, Yukui Rui

**Affiliations:** 1 Beijing Key Laboratory of Farmland Soil Pollution Prevention and Remediation, College of Resources and Environmental Sciences, China Agricultural University, Beijing 100193, China; xtang12@126.com (Y.T.); zgk18451176676@163.com (G.Z.); 2 State Key Laboratory for Environmental Chemistry and Ecotoxicology, Research Center for Eco-Environmental Sciences, Chinese Academy of Sciences, Beijing 100085, China; wczhao_st@rcees.ac.cn (W.Z.); zqtan@rcees.ac.cn (Z.T.); 3 Jiaer Chen Academician Workstation, Jinan Huaxin Automation Engineering Co., Ltd., Xincheng Road, Shanghe County, Jinan 251616, China; 15954699400@163.com; 4 Department of Environmental Science and Engineering, University of Science and Technology of China, Hefei 230026, China; zhangpeng1987@ustc.edu.cn; 5 State Key Laboratory for Biology of Plant Disease and Insect Pests, Institute of Plant Protection, Chinese Academy of Agricultural Sciences, Beijing 100193, China

**Keywords:** nanotechnology, nano-fertilizers, nano-pesticides, food security, sustainable agriculture

## Abstract

Nanotechnology emerges as an important way to safeguard global food security amid the escalating challenges posed by the expansion of the global population and the impacts of climate change. The perfect fusion of this breakthrough technology with traditional agriculture promises to revolutionize the way agriculture is traditionally practiced and provide effective solutions to the myriad of challenges in agriculture. Particularly noteworthy are the applications of nano-fertilizers and pesticides in agriculture, which have become milestones in sustainable agriculture and offer lasting alternatives to traditional methods. This review meticulously explores the key role of nano-fertilizers and pesticides in advancing sustainable agriculture. By focusing on the dynamic development of nanotechnology in the field of sustainable agriculture and its ability to address the overarching issue of global food security, this review aims to shed light on the transformative potential of nanotechnology to pave the way for a more resilient and sustainable future for agriculture.

## 1. Introduction

The world’s agricultural sector has advanced remarkably in the previous several decades. Thanks to technological innovations and improved agricultural practices, we have produced more food to fulfill our population’s demands. However, by the end of the century, there will be nine billion people on the planet [1], posing a significant challenge to food supply and it also means global food demand will increase further over the next 40 years. Recent studies suggest that by 2050, world food demand will increase by 70 to 100 percent [2,3]. At the same time, droughts, floods, and severe temperatures caused by climate change all pose a threat to agricultural development and production, further exacerbating food security concerns [4].

With the world’s population rapidly increasing, the changing climate, and the increasing scarcity of natural resources, improving food production and quality has become a top priority [5]. At the same time, addressing the effects of the environment on the food chain is urgently necessary, including increased competition for land and energy, in addition to overfishing, which influence our capacity to produce food [6]. A lot of focus has been placed on finding sustainable ways to meet the world’s food needs in recent years.

**Figure 1:**
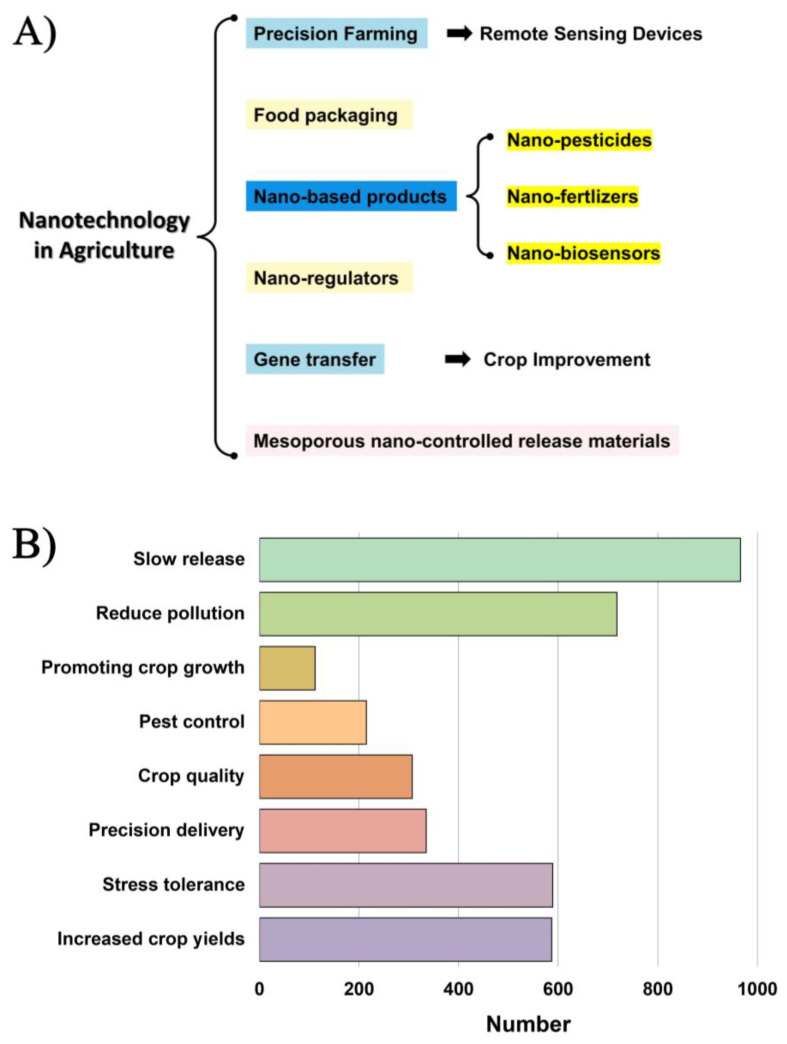
(**A**) Applications of nanotechnology in agriculture (drawn using Microsoft Office PowerPoint 2019). (**B**) Various roles played by nanotechnology in agricultural production since 2000 were searched through the Web of Science (drawn using Origin 2023b).

In this environment, food security and sustainable agricultural growth empowered by nanotechnology have become new research hotspots. The emergence of nanotechnology brings new hope for sustainable agricultural production and is expected to transform traditional agriculture into precision and smart agriculture [7]. Figure 1A illustrates the utilization of nanotechnology in farming. Since 2000, Figure 1B illustrates the numerous roles performed by nanotechnology in productivity in farming. The use of nanotechnology in agriculture, especially nano-pesticides and fertilizers [8], has opened up new paths for sustainability in agriculture.

In this paper, we introduce nano-fertilizers and nano-pesticides, analyze their advantages over traditional agrochemicals, and draw out the challenges nanotechnology faces in agricultural applications. Through comprehensive analysis, we offer several potential research avenues for advancing nanotechnology’s involvement in sustainable farming. Therefore, contributing to the attainment of sustainable production of food and global security of food.

## 2. Overview of Nanotechnology

### 2.1. Definition and Basic Concepts of Nanotechnology

**Figure 2:**
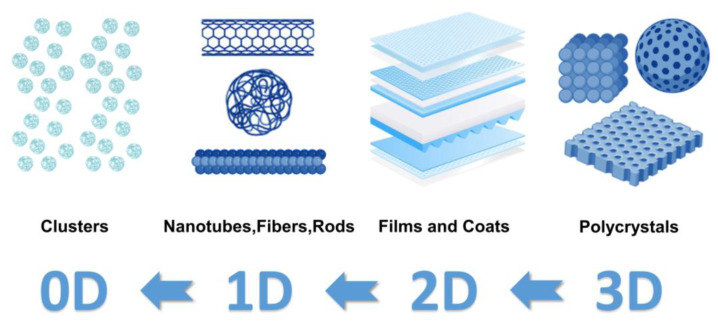
Dimension-based classification of nanomaterials (drawn using the bioRender website https://app.biorender.com/, accessed on 1 December 2023).

The term “nanotechnology” is frequently used to characterize structures made of tiny pieces that are assembled in bottom-up or top-down ways to form structures as large as hundreds of nanometers [9,10]. The strictest definition of nanotechnology provided by the National Nanotechnology Initiative relates to structures with at least one dimension of between one and one hundred nanometers [11]. There are many types of nanomaterials, which can be categorized according to the material matrix: metal-based nanomaterials, carbon-based nanomaterials, nanocomposites, and semiconductor nanomaterials [12]. Dimensions can be categorized into four categories 0D, 1D, 2D, and 3D (D stands for dimension) as shown in Figure 2 [13].

Nowadays, nanotechnology is used in many industries, including agriculture [14], environmental protection [15], food processing and packaging [16], textile [17], and construction [18], among others, posing fresh possibilities and difficulties for established industrial technology. The application of nano pesticides and fertilizers in agriculture is where nanotechnology is most visible [8,19]. Because of their unique properties, which include a large surface and controlled release [20], nanomaterials are perfect for increasing agricultural output.

### 2.2. Preparation and Characterization of Nanomaterials

**Figure 3:**
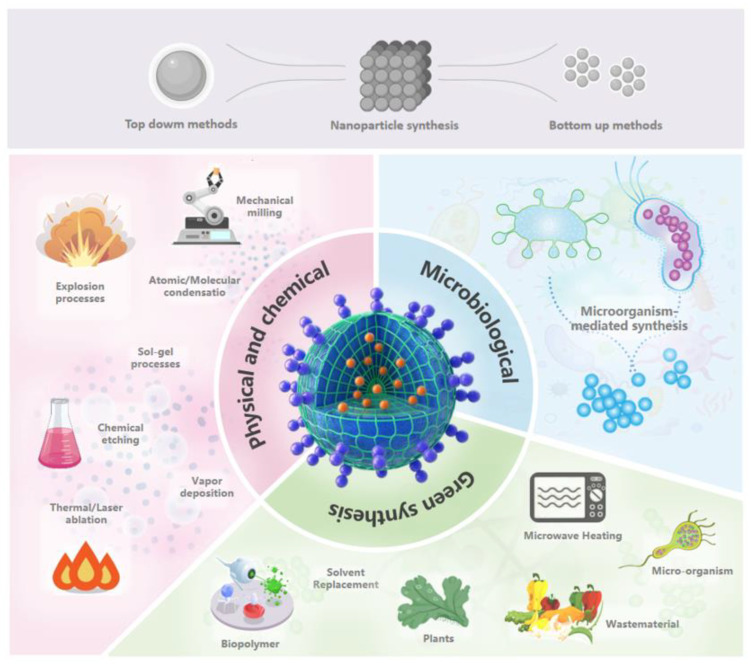
Synthesis methods of nanomaterials (drawn using Microsoft Office PowerPoint 2019).

Nanomaterials are usually prepared by “top-down” or “bottom-up” methods, which can also be categorized as physical and chemical methods, microbial-mediated synthesis, and green synthesis (Figure 3) [21]. The technique of choice is determined by the kind of nanomaterials sought, their properties, and their applications. Physical synthesis methods are suitable for materials with high purity and crystallinity, chemical synthesis methods offer versatility and controllability [22], microbial-mediated synthesis methods are biocompatible, and green synthesis methods emphasize environmental protection and sustainability [12]. Table 1 shows their main differences as well as their application areas. Among them, the microbial (fungi, bacteria, viruses, etc.) mediated synthesis of nanomaterials using microorganisms under artificial conditions at an ecological pace is more advantageous [23].

### 2.3. Nanotechnology in Agriculture

In the last decade, nanotechnology has ushered in a wave of transformative energies and pioneering innovations in agriculture. The implementation of nano-fertilizers stands out as a notable success, enhancing nutrient efficiency in crops and concurrently curbing the overuse of chemical fertilizers [24]. This not only elevates crop yields but also addresses concerns related to fertilizer waste [19]. The widespread adoption of nano-pesticides marks another milestone, effectively mitigating environmental pollution and minimizing adverse effects on ecosystems.

For example, carbon nanotubes in one-dimensional nanomaterials have been shown to enhance plant growth by improving nutrient use efficiency [25], and are therefore widely used in pesticides and fertilizers. Carbon nanotubes usually require physicochemical characterization before use by using scanning electron microscopy (SEM) and transmission electron microscopy (TEM) to observe the morphology and dimensions of CNTs; by using techniques such as Fourier transform infrared spectroscopy (FTIR) and X-ray photoelectron spectroscopy (XPS) to analyze the surface chemistry of the CNTs; and by using spectroscopy or mass spectrometry to accurately determine the concentration of CNTs [26]. However, their cytotoxicity needs to be considered before application. Pulskamp et al. showed that carbon nanotubes did not show acute toxicity but induced intracellular reactive oxygen species, which was associated with contaminants [27]. Dumorter et al. reported that CNTs could be taken up by lymphocytes and macrophages in vitro without affecting cell viability, that CNTs did not affect immune cell functional activity [28]. Khodakovskaya et al. concluded that high concentrations of carbon nanotubes affect microbial communities, while low concentrations have insignificant effects [25].

As a representative two-dimensional nanomaterial, graphene demonstrates significant plant-promoting effects and has been widely used in agriculture, both as an insecticide and as a fertilizer [29]. Graphene also needs to be characterized before use by detailed structural and surface morphology characterization using techniques such as Raman spectroscopy and atomic force microscopy (AFM); investigating the surface chemistry of graphene using methods such as XPS and nuclear magnetic resonance spectroscopy (NMR) [30]; and evaluating graphene at different concentrations through biological experiments for biological effects on plants and microorganisms. Through their study, Begum et al. found that graphene-induced production of excess ROS inhibits plant growth, reduces biomass, and has toxic effects on terrestrial plant species such as cabbage and tomato [31]. Similarly, a study by Du et al. found that graphene oxide significantly altered the structure and composition of soil bacterial communities [32].

Metal oxide nanoparticles, such as mesoporous silica, which is a three-dimensional nanomaterial, have the potential to modulate nutrient release and therefore have been widely studied for use as pesticides and fertilizers [33]. Characterization methods for metal oxide nanoparticles include observation of the particle size and morphology of metal oxide nanoparticles using techniques such as TEM and dynamic light scattering (DLS); analysis of the structure and surface composition of metal oxide nanoparticles using means such as X-ray diffraction (XRD) and XPS [34]; and evaluation of the potential impacts of metal oxide nanoparticles on soil ecosystems through soil microbial activity measurement as well as gene expression analysis and other methods to assess the potential impact of metal oxide nanoparticles on soil ecosystems. However, high concentrations of metal oxide nanoparticles, such as CuO and ZnO, may have toxic effects on beneficial microbial communities in the soil, leading to bacterial or fungal inhibition [35].

Furthermore, the integration of nanotechnology into plant genetic improvement has revolutionized the precision with which scientists can edit and regulate plant genes [36]. This precision breeding enhances traits such as disease resistance, drought tolerance, and overall crop yields. Simultaneously, nanotechnology has catalyzed advancements in agricultural informatization. The incorporation of nano-sensors and monitoring technology empowers farmers to engage in real-time monitoring of soil quality, plant conditions, and weather patterns [37]. This scientific management approach not only boosts the efficiency of agricultural production but also reduces resource wastage, epitomizing the aspirations of precision agriculture.

Nanotechnology’s advent expands the horizons of traditional agricultural production, offering crops the capacity to adapt to diverse environmental conditions. This heightened adaptability contributes to increased resilience within agricultural systems. The successful incorporation of nanotechnology in agriculture provides robust support for augmenting food production, enhancing agricultural sustainability, and mitigating environmental impacts. These innovations, thus, present new avenues for the future of agriculture, playing a pivotal role in bolstering global food security and promoting the principles of sustainable agriculture.

## 3. Nano-Fertilizers in Farming

### 3.1. Types of Nano-Fertilizers

Nano-fertilizers, emerging from advancements in nanotechnology, exhibit diverse properties catering to specific nutrient requirements. They are mainly divided into macro-nutrient element nano-fertilizers and micro-nutrient element nano-fertilizers in terms of nutrient elements. For macro-nutrient elements, such as nitrogen, phosphorus, and potassium, processing them into nanoscale may change their bio-effectiveness as well as the way of crop absorption [38]. Taking the macronutrient element nitrogen as an example, nano-nitrogen fertilizer can improve the diffusion speed of nitrogen in the soil, improve its effectiveness, and improve the efficiency of plant nitrogen absorption [39]. By changing the pH of the inter-root microenvironment, nano-fertilizers can increase root absorption of these macronutrients. Micronutrients have an important impact on disease resistance in crops [40]. Nanoscale processing of boron and molybdenum can improve their solubility and exchangeability in the soil, making them more readily available for plant uptake, which in turn improves crop resilience [41]. In terms of mechanism of action, there are two primary types: The first relates to nanomaterials that convert fertilizers to the nanoscale to provide plants with one or more nutrients to aid in development and productivity. Second, although not directly feeding crops, the use of engineered nanomaterials as fertilizer transporters improves the effectiveness of traditional fertilizers and enables targeted fertilizer transport or release management.

The mechanism of nanoparticle transport in plants involves two main pathways, root uptake and foliar uptake. In root uptake, nano-fertilizers may enter plant cells by penetrating root cell membranes, through translocation channels or ion channels. Factors such as ion concentration, soil pH, and temperature can all have an impact on this process. Conversely, in foliar uptake, nanoparticles may enter the leaf by stomata or trichome roots [42], or by penetrating the cell membrane of the leaf and transferring it to the tissues to achieve elemental uptake. This approach may have a more direct impact on plant growth because foliar uptake is closely related to physiological processes such as photosynthesis. The deposition of nanoparticles onto photosynthetic surfaces can induce thermal effects on foliage, potentially leading to the obstruction of stomata and subsequent disruption of gas exchange. Consequently, this perturbation can elicit modifications in the plant’s physiological and cellular processes [43].

Overall, nano-fertilizers are supplied through root or foliar application, then transferred to the above-ground part via the root endodermis and epidermis, or taken up by leaf pores and carried via the phloem [44]. Nanoparticles can penetrate plant cells directly via the cell wall structure and reach the plasma membrane when they are smaller than the cell membrane particle sizes (5–20 nm) [45,46]. When nanoparticles attach to various cytoplasmic organelles, metabolic activities can be interfered with [47]. Engagement with designed nanoparticles has the potential to enlarge pores or induce new cell wall pores, hence increasing nanoparticle absorption.

**Figure 4:**
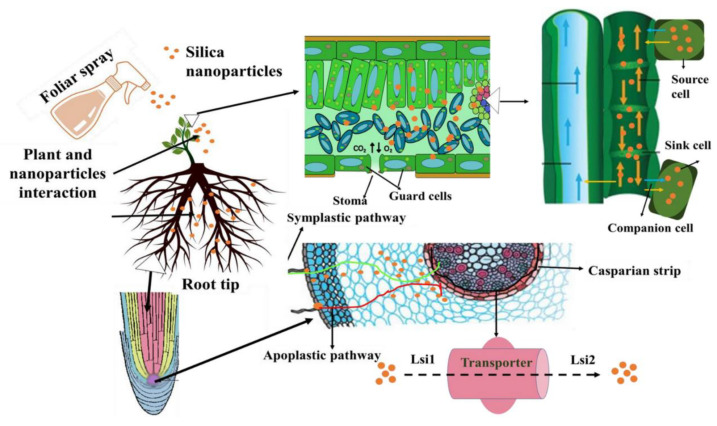
Silica nanomaterial transport in plants [48].

The transport of silica nanoparticles in crops has been reported (Figure 4), where the nanoparticles are internalized mainly through two pathways, i.e., extracellular and intracellular membranes. Additionally, it interacts with the root system of plants, passing via the Casparian strip, endodermis, and epidermis before entering the xylem through transporter proteins such as Lsi1 [48]. However, in many circumstances, silica nanoparticles enter the plant via the stomata and are then transported via the exosome route [49].

Some studies report various hypotheses to explain the mechanism of action. For instance, irrigation is the best way to apply nanoparticles if they enter the plants through the xylem [50]. Additionally, foliar spraying is the best approach to prevent nanoparticles from migrating via the phloem [51].

### 3.2. Application of Nano-Nitrogen Fertilizer

The utilization of nano-nitrogen fertilizers exhibits noteworthy advantages in augmenting nitrogen use efficiency [39]. Owing to their diminutive particle size, nano-nitrogen fertilizers exhibit enhanced binding to soil particles, prolonging the retention time of nitrogen in the soil. This attribute serves to mitigate volatilization and leaching losses of nitrogen. The precise and targeted release mechanism associated with nano-nitrogen fertilizers contributes to more efficient uptake of nitrogen by plants during critical growth stages [24], thereby minimizing nitrogen waste.

Moreover, the size effect of nano-nitrogen fertilizers assumes a pivotal role in inter-root interactions. The reduced size facilitates easier penetration of the plant root system, enhancing the efficiency of nitrogen uptake [52]. This not only diminishes the requirement for nitrogen fertilizers and amplifies nitrogen utilization by plants but also curtails the adverse environmental impact, concurrently safeguarding agricultural yields [53]. In comparison to traditional nitrogen fertilizers, nano-nitrogen fertilizers exhibit notable advantages in enhancing crop yield and quality [54]. The precision of the release mechanism in nano-nitrogen fertilizers results in a more consistent and even supply of nitrogen, contributing to the maintenance of stable nitrogen levels in crops throughout the growth cycle. This is anticipated to mitigate the issue of nitrogen oversupply associated with conventional nitrogen fertilizers, thereby addressing environmental concerns.

Furthermore, owing to the efficient uptake characteristics of nano-nitrogen fertilizers, plants demonstrate enhanced nitrogen utilization, leading to improved growth and yield. This development is poised to offer more sustainable nitrogen management solutions for agricultural production, presenting farmers with smarter and environmentally-friendly options. In summary, nano-nitrogen fertilizers exhibit evident advantages in augmenting nitrogen use efficiency, mitigating environmental pollution, and enhancing crop yield and quality [50]. These attributes introduce novel possibilities for sustainable agricultural development.

### 3.3. Effectiveness of Nano Phosphate Fertilizer

Nano phosphorus fertilizers exhibit distinctive outcomes in amplifying the effectiveness of phosphorus in the soil. The nanoscale particles possess a substantial specific surface area, facilitating the facile combination of nano phosphorus fertilizer with soil particles to form a more stable complex [55]. This process retards the migration and leaching of phosphorus in the soil, thereby heightening its effectiveness. Additionally, the nano-size property intensifies the interaction between the nano phosphorus fertilizer and the plant root system, fostering the efficiency of phosphorus uptake by the plant.

Nano phosphate fertilizers demonstrate a positive impact on promoting plant growth for agricultural production [56]. By augmenting the effectiveness of phosphorus in the soil, nano phosphorus fertilizers provide a more readily available nutrient source for plants, consequently expediting the growth process. This growth promotion is manifested in increased plant height, expanded leaf area, and a more developed root system, ultimately leading to higher yields [55]. Notably, this growth-promoting effect exhibits a degree of consistency across various soil types and climatic conditions.

### 3.4. Characteristics of Nano Potassium Fertilizer

The utilization of potash nanoparticles exerts a significant influence on plant resilience. Nano-sized particles exhibit increased penetrative capability into the plant root system, thereby facilitating a more efficient delivery of potassium nutrients [52]. This enhancement contributes to bolstering plant resistance against various adverse conditions, including, but not limited to, drought, salt, and diseases. By elevating the physiological activity and nutrient balance of the plant, potassium nano-fertilizers fortify its resilience to challenging environmental conditions, ultimately enhancing overall crop survival and yield [56].

In comparison with traditional potash fertilizers, nano-potash fertilizers manifest more pronounced effects under diverse environmental conditions. A comprehensive understanding of the superiority of nano-potash fertilizers can be gained by evaluating their performance across different soil types, humidity levels, and temperatures. This comparative analysis holds practical significance in guiding farmers toward the most suitable fertilizer options tailored to specific environmental conditions.

### 3.5. Application Cases

The application cases of nano-fertilizers on major crops such as rice and wheat provide compelling evidence [57]. Practical implementations of nano-fertilizers have yielded substantial impacts on both yield and quality. For instance, in rice cultivation, the use of nano-fertilizers has markedly increased grain weight and improved overall grain quality [58]. The assessment of nano-fertilizer sustainability in agricultural systems necessitates long-term studies. These studies should not only focus on yield performance within a single season but also track the effects of nano-fertilizers across multiple growing cycles. Observations over an extended period can offer a more comprehensive understanding of the potential long-term impacts of nano-fertilizers on soil quality, plant growth, and environmental considerations. Cumulatively, these application cases provide robust support for the adoption of nano-fertilizers in agriculture, contributing positively to increased crop yields, enhanced quality, and the realization of agricultural sustainability.

### 3.6. Influencing Factors

The type, dose, method, and structure of nano-fertilizers all have an impact on the absorption, transformation, transport, and accumulation of nanoparticles by crop leaves or roots [5,59]. Plant translocation processes differ in nano-fertilizer aggregation, dispersion, absorption, bioavailability, and translocation mechanisms [60,61]. In addition, plant species differences and the zeta potential of nanoparticles are factors that affect it, along with the structure, pH, and texture of the soil [62]. The stability and solubility of nanoparticles can be affected by pH variations in the soil, affecting the rate of nutrient release. The distribution and movement of nanoparticles in soil may be influenced by soil structure and texture. Plant species variations may result in variances in nano-fertilizer absorption and response. The capacity of nanoparticles to penetrate the chloroplast membrane is significantly related to their zeta energy value [63]. Some research indicates that the same nanoparticles may affect various sections of the same crop differently [64].

**Figure 5:**
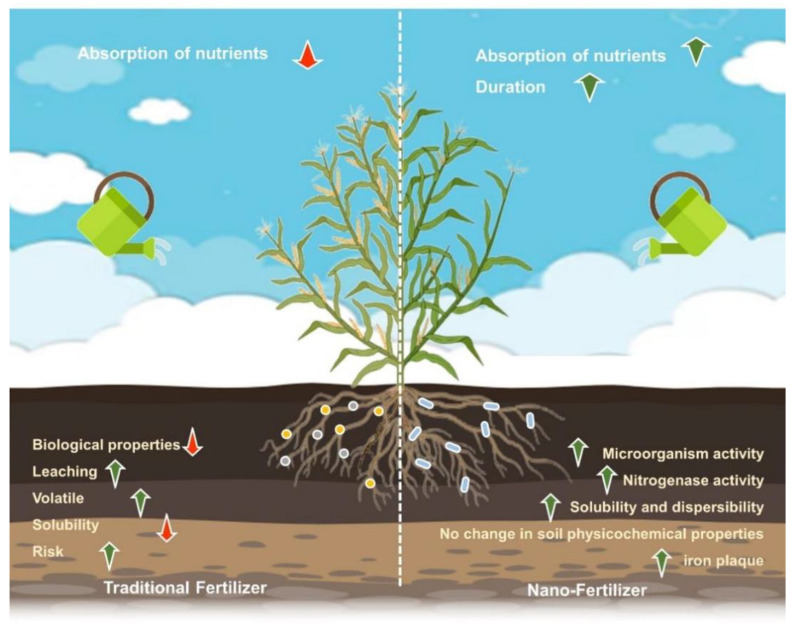
Comparison of nano-fertilizers with conventional fertilizers (drawn using bioRender website https://app.biorender.com/, accessed on 1 December 2023).

Nano-fertilizers, as an innovative agricultural technology, have multiple advantages when compared with traditional agricultural fertilizers (Figure 5). These include precise control of nutrient release [24], improved soil nutrient use efficiency [24,65], boosted crop output, significantly reduced environmental pollution [66] and sustainably released fertilizers, as well as prevention of unwanted interactions between microorganisms [67], improved tolerance mechanisms within plants to multiple environmental stresses, enhanced plant metabolism [68] and additional advantages for sustainable agriculture when used in combination with microorganisms, etc. [69]. It is anticipated that the use of nano-fertilizers will significantly affect agricultural productivity globally [5].

By delving into these influencing factors and recognizing the complexities involved in the application of nano-fertilizers, researchers, and practitioners can innovate and improve accordingly to ensure that their potential benefits are maximized and their negative impacts are minimized. In summary, a comprehensive exploration of all aspects related to nano-fertilizers, from their types and mechanisms of action to their specific applications and influencing factors, can help to provide a deeper understanding of the enormous potential of nano-fertilizers in sustainable agricultural development and can help to provide an avenue for promoting more resilient and environmentally friendly approaches to food production.

## 4. Nano-Pesticides in Agriculture

The integration of nanotechnology into the realm of pesticides presents innovative solutions for conventional agricultural practices. Incorporating nanotechnology into the pesticide formulation process holds the promise of significantly enhancing pesticide effectiveness while concurrently mitigating adverse environmental impacts. Nano-pesticides, defined as formulations or products containing engineered nanoparticles with biocidal capabilities [70].

Nano-pesticides come in a variety of forms, such as lipids, polymers, and metal-organic frameworks. These materials exhibit different mechanisms in pesticide delivery and release. Polymer-based nano-pesticides may achieve precise control of pesticide attachment and release on crop surfaces through the tunability of their microstructures [71]. On the other hand, lipid-based nano-pesticides may enhance persistence and stability by allowing for the gradual release of pesticides within the water column due to the lipid layer’s solubility [52]. Metal-organic frameworks, on the other hand, may realize efficient loading and gradual release of pesticides through their highly ordered pore structure. Many nano-pesticide compounds have been demonstrated to be more effective than their commercial equivalents [72].

### 4.1. Fundamentals of Nano-Pesticides

In the formulation of nano-pesticides, nanotechnology is frequently employed to manipulate the particle size and structure of pesticides, often achieved through the encapsulation of the active ingredient in nanoparticles [73]. This approach enhances the stability and persistence of pesticides, prolonging their presence on the plant surface. Such application methods contribute to increased resistance against washout in adverse climate conditions, consequently augmenting their efficacy. Certain nanocarriers exhibit responsiveness to environmental changes, affording improved targeting capabilities [74].

The distinctive properties of nanoparticles in pesticides offer unique advantages for plant protection applications [75]. The substantial increase in surface area facilitates enhanced interaction between nano-pesticides and the plant surface, improving adhesion effects. Furthermore, the improved penetration ability of nanoparticles not only accelerates pesticide delivery but also enables deeper penetration into plant tissues, resulting in more comprehensive control effects [52].

Through the application of nanotechnology, precise control over pesticide dosage becomes achievable, leading to reduced pesticide usage while maintaining efficient control [76]. The heightened permeability and precise release mechanisms of nano-pesticides enable even relatively small doses to maximize their effects on plants. This contributes to minimizing the negative environmental impacts of pesticides, reducing costs in agricultural production, and enhancing control sustainability. These unique properties of nanoparticles form a robust foundation for optimizing the efficacy of pesticides.

### 4.2. Pest Control Mechanisms

The mechanisms by which nano-pesticides affect pests are complex and diverse [48]. Through mesoporous properties, nano-pesticides may form tiny traps on crop surfaces, increasing adsorption and attachment to pests. Modulation of solubility may lead to a more gradual release of the pesticide in the pest, increasing the toxicity to the pest. The mode of release of active ingredients, such as the surface reactivity of nanocarriers, may allow pesticides to act in a more targeted manner on pest physiological systems, increasing their lethal effects. Smaller-sized nanoparticles exhibit enhanced penetration capabilities through both the epidermis and roots of plants, facilitating broader distribution of pesticides within the plant and thereby augmenting their efficacy against pests [77]. The larger surface area of nanoparticles contributes to improved adhesion of pesticides to plant surfaces, extending their duration of action and diminishing the necessity for frequent pesticide applications.

Some nano-pesticides are specifically engineered for precise delivery to pest-affected areas through targeted delivery systems. This targeted approach minimizes the impact on non-target organisms, concurrently enhancing the pesticide’s effectiveness.

The inherent properties of nanomaterials enable the design of systems for controlled pesticide release. For instance, the response to environmental factors such as humidity, temperature, pH, etc., allows the regulation of pesticide release as required. This controlled release mechanism not only improves pesticide effectiveness but also reduces wastage, contributing to more sustainable and efficient pesticide applications.

For example, silica nanoparticles are used as insecticides in two main ways: direct application of silica nanoparticles to crops to generate a silica coating that hinders insect and larval development and entrance, and use of mesoporous silica nanoparticles to distribute commercial insecticides [78]. Because silica has a drying effect, direct application is more lethal to adults and their larvae. Silica nanoparticles also block the trachea or stomata of insects, which can be fatal to insects [48].

### 4.3. Advantages of Nano-Pesticides

**Figure 6:**
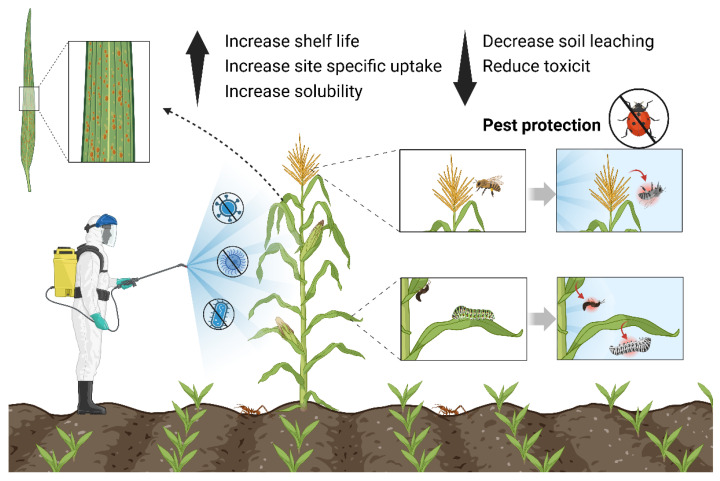
Role of nano-pesticides and their benefits (drawn using bioRender website https://app.biorender.com/, accessed on 1 December 2023).

Nano-pesticides, as an innovative technology, have multiple advantages in agriculture (Figure 6).

Nano-pesticides have better environmental friendliness compared to conventional pesticides. The advantage of their small scale will reduce the non-specific effects on beneficial insects and microorganisms [8], which improves the protective effects on crops. In addition, nano-pesticides can accurately control pest and disease transmission while reducing the number of pesticides applied, improving the efficiency of utilization, which is expected to reduce the risk of contamination of soil and water bodies. Controlled release kinetics; enhanced permeability, stability, and solubility; extended pesticide persistence; increased efficacy, and prevention of premature degradation of the active ingredient under stress conditions are all possible with nano-encapsulated pesticides, potentially leading to improved pest control efficiency [79]. Nano-pesticides have also been shown to have significant effects in improving crop yields compared to conventional insecticides [80].

In addition, the application of chiral nanomaterials in nano-pesticides is one of the hotspots of current research [8]. They may achieve efficient control of specific pests through chiral recognition and selective adsorption. They may offer superior biocompatibility, less toxicity to non-target species, and better fulfillment of environmental friendliness and sustainability standards [81,82]. In conclusion, the application of nanotechnology to pesticides is innovative, and ongoing research on nano-pesticides will continue to present opportunities for sustainable pest management and increased agricultural productivity.

## 5. Current Gaps of Nano Agrochemicals

While nanotechnology brings many innovative solutions to modern agriculture, there are numerous challenges to be faced.

The cost of manufacturing and the problem of sustainability are the main obstacles faced in the preparation of nano-agricultural products. The high costs associated with nanotechnology and materials impede their widespread use. Collaborations between research institutions, governments, and industry are essential to overcome this challenge. Financial incentives, research grants, and partnerships can facilitate cost-reduction measures and make nano-agrochemicals more accessible to a wider range of agricultural practitioners. Sustainability is another key issue. The production process of nanoproducts must prioritize ecological goodness. Adopting green and sustainable methods in the production of nanomaterials can mitigate negative environmental impacts. Researchers and industry stakeholders should explore eco-friendly manufacturing processes and materials to ensure the long-term sustainability of nano-agrochemicals.

Second, because metal-based nanoparticles may dissolve and release metal ions into the atmosphere [83], it is not yet fully clear how long-term use of nanomaterials in agriculture could affect soil and ecosystems, but the risk exists [84]. More research is needed to ascertain their potential effects on soil and ecosystems with increased research efforts to assess and quantify these potential risks. Collaborative research involving environmental scientists, agronomists, and ecologists can contribute to a clearer understanding of the environmental impacts of nano-agrochemicals.

Safety and regulatory issues are also a prominent challenge. Health risk evaluations and effective regulatory frameworks are needed to ensure that their use in agriculture is safe for farmers, consumers, and the environment. Government agencies and international organizations should cooperate to develop standards and regulations for the use of nano-agrochemicals.

Furthermore, programs for education and training are also necessary to optimize the advantages of nanotechnology while reducing any potential risks. Technology diffusion and training of agricultural practitioners are key to ensuring that agricultural practitioners can use nanotechnology properly. Increased investment in technology diffusion and training activities will ensure that practitioners can utilize nanoproducts responsibly, which in turn will improve the overall effectiveness and safety of nano-agrochemical applications.

Social awareness and acceptance are also issues that need to be addressed, as consumers often associate food and agricultural products with “natural”, “organic”, “healthy” and “environmentally friendly” labels. “Environmentally friendly”, in the application of nanotechnology may be subject to public skepticism and concern [85]. Promoting public understanding of the benefits and safety measures of nano-agrochemicals is therefore essential. Activities such as open dialogues and presentations can dispel misconceptions and increase public acceptance. In addition, the development of sound regulations and ethical standards are the cornerstones of ensuring the safe and sustainable development of nanotechnologies. Governments and regulatory agencies should cooperate to establish clear guidelines for the production, distribution, and use of nano-agrochemicals. Active participation in international cooperation can facilitate the development of global norms and standards and promote responsible agricultural nanotechnology practices on a global scale.

## 6. Future Outlook

The future development of nanotechnology in agriculture holds great potential. To fully realize its potential, the future development of nanotechnology applications in agriculture should focus on the following aspects:

1. Precision agriculture and smart agriculture innovation [86]:

Utilizing advanced sensing technologies, big data analyses, and artificial intelligence to achieve smarter agricultural production [86]. Development of agricultural robots with autonomous decision-making capabilities capable of making appropriate farm management decisions in real-time environments. For example, nanorobots with high-speed wireless connectivity [87], can scan and analyze plant capillaries [88]. Integrating nanorobots into agriculture has the potential to revolutionize modern agricultural production methods. Integration of nano-pesticides, nano-fertilizers, and nano-biosensing technologies into multifunctional nano-agricultural systems is crucial. This facilitates real-time monitoring and regulation of agricultural production processes and can increase efficiency, reduce resource use, and improve the sustainability of agricultural practices [89].

2. Innovations in sustainable nano-pesticide and fertilizer design:

Focus on designing eco-friendly and biocompatible nano-pesticides to reduce the impact on non-target organisms and minimize negative impact on the environment. Research on intelligent design and release control of nano-pesticides to meet the varying needs of different crops and adapt to changing environmental conditions [90]. Develop responsive nano-pesticides that can be released at specific plant growth stages or environmental conditions to enhance effectiveness and minimize negative impacts on the environment. Considering the incorporation of nano-catalysts [91] could further improve efficiency, reduce usage, and minimize environmental impacts. The research and development of nano-fertilizers is promoted to achieve more precise and efficient nutrient release and reduce the pollution of soil and water bodies. At the same time, its interaction with the plant root system can be studied in depth to promote more efficient nutrient uptake and meet the goal of maximizing agricultural productivity with minimal environmental impact.

3. Gene editing combined with nanotechnology:

Nanotechnology is used to optimize gene editing tools [88] and improve the precise editing of crop genes. Exploring nanobiotechnology from a genomic perspective opens up new avenues for modifying plant genes, which has the potential to improve plant resistance and yield [92]. Explore the potential of nanocarriers for plant gene delivery and expression [93,94] to improve plant resistance to adversity. This provides an important avenue to improve crop quality and yield and to address agricultural challenges.

4. Social participation and education:

To ensure wide acceptance and practical application of nano-agricultural technologies, it is important to deepen collaboration with agricultural practitioners and communities to ensure that the application of nano-agricultural technologies meets local needs and sustainable development goals. Wide-ranging social awareness and education campaigns should be conducted to promote a better public understanding of the role of nanotechnology in agriculture and to eliminate negative perceptions.

5. Global cooperation and standard setting:

Actively participate in international cooperation to develop global standards and ethical norms for nano-agricultural technologies. Promote global research programs on sustainable agriculture, share best practices and scientific research results, and facilitate transnational cooperation in agricultural innovation.

6. Biosynthesis and sustainable production:

Explore the use of biosynthetic methods, such as plant extracts, as an alternative to traditional physical or chemical synthesis processes to prepare nanomaterials in a more environmentally friendly way [88]. Promote research on the preparation of metal nanoparticles by biological methods to achieve large-scale production while reducing environmental impact [23]. Investigating these avenues can help develop green and sustainable large-scale production processes for metal nanoparticles.

7. Nanotechnology and climate change adaptation:

Utilize nanotechnology innovations to improve crop adaptation to climate change and enhance resistance to drought, salinity, and other adversities. Explore the application of nanomaterials in water resource management, soil improvement, etc., to help sustainable agricultural development.

The prospects of agricultural nanotechnology depend on innovative approaches, sustainable practices, and collaborative efforts. By combining technological innovation with ethical guidelines, as well as global collaboration, we can pave the way for a more resilient, efficient, and sustainable agricultural future, suggesting possible solutions to future food security issues.

## Figures and Tables

**Table 1:** Differences in the synthesis of nanomaterials by different methods, and applications of different methods.

Classification	Method	Differences	Applications	Cases
Physical synthesis	Vapor deposition, sputtering	Does not involve chemical reactions and relies heavily on physical processes	Preparation of nanomaterials with high crystallinity and purity, such as nanofilms, nanowires and nanoparticles	Nano-fertilizers
Chemical synthesis	Solution methods, hydrothermal synthesis	Involves a chemical reaction, usually requiring solvents, catalysts, specific reaction conditions	Suitable for the preparation of nanomaterials with high purity and high crystallinity	Biosensors, nanodrug delivery, nano-pesticides
Bio-synthesis	Microbes, plants or biomolecules	Involves bio-reductive or biosynthetic processes that are defined by the biology of the biological system and the growing circumstances.	Can be used in biomedical applications with natural biocompatibility	Nano drug carriers, biosensors

## Data Availability

No new data were created or analyzed in this study. Data sharing is not applicable to this article.
